# Immunoregulation: the interplay between metabolism and redox homeostasis

**DOI:** 10.3389/frtra.2023.1283275

**Published:** 2023-11-27

**Authors:** E. Perpiñán, A. Sanchez-Fueyo, N. Safinia

**Affiliations:** Department of Inflammation Biology, School of Immunology and Microbial Sciences, Institute of Liver Studies, James Black Centre, King’s College London, London, United Kingdom

**Keywords:** regulatory T cells, reactive oxygen species, anti-oxidants, NRF2, mTOR

## Abstract

Regulatory T cells are fundamental for the induction and maintenance of immune homeostasis, with their dysfunction resulting in uncontrolled immune responses and tissue destruction predisposing to autoimmunity, transplant rejection and several inflammatory and metabolic disorders. Recent discoveries have demonstrated that metabolic processes and mitochondrial function are critical for the appropriate functioning of these cells in health, with their metabolic adaptation, influenced by microenvironmental factors, seen in several pathological processes. Upon activation regulatory T cells rearrange their oxidation-reduction (redox) system, which in turn supports their metabolic reprogramming, adding a layer of complexity to our understanding of cellular metabolism. Here we review the literature surrounding redox homeostasis and metabolism of regulatory T cells to highlight new mechanistic insights of these interlinked pathways in immune regulation.

## Introduction

Oxidative stress, teamed with heightened levels of inflammation and metabolic disturbances, underlies the pathology of many conditions, including cardiovascular diseases, cancer, autoimmune and neurodegenerative disorders (1–4). This phenomenon is the result of either an overproduction of reactive oxygen species (ROS) and/or an imbalance between production of oxidants and antioxidants defenses (5). However, redox states have an important role in immunity and T cell function. It is well known that moderate levels of ROS are essential as pivotal second messengers for T cell receptor signaling and activation and participate in chemotaxis and antigen cross-presentation (6–8), whilst elevated levels of ROS have a detrimental effect on immune regulation (9, 10), causing damage of proteins, DNA and carbohydrates.

Under inflammatory conditions, regulatory T cells (Tregs), a subset of immune cells, play an important role at curbing excessive immune activation and maintaining immune homeostasis (11). With the growing appreciation of the relationship between metabolism and immunity, we now realise that the phenotypic stability, suppressive function and survival of Tregs are tightly determined by specific metabolic requirements and programs, with their metabolic adaptation seen in various pathological conditions. Previous studies, mainly based in murine models, have described the engagement of different metabolic pathways by Tregs to exert their function. Unlike effector T cells (Teff) that use aerobic glycolysis to meet their bioenergetic demands under steady state conditions, Tregs largely rely on mitochondrial metabolism through a selective dependency on fatty acid oxidation to sustain the tricarboxylic acid (TCA) cycle and oxidative phosphorylation (OXPHOS) reactions (12, 13). Likewise, Tregs show a distinct metabolic profile during proliferation, migration, and suppressive functions. Following stimulation, Treg proliferation is dependent on an oscillatory switch of glycolysis dictated by increased activity of the PI3K/Akt and mTOR pathways at the early phase (14). However, in contrast to Teff, Tregs also use an array of substrates from the fatty acid oxidation and amino acid metabolism to fuel the TCA cycle. During migration, Tregs engage in glycolysis like most migratory cells (15). Although glycolysis promotes Treg growth and motility, the immunosuppressive function and stability of Tregs is compromised. To exert their suppressive functions, Tregs require a stable FOXP3 expression which in turn controls the induction of OXPHOS activity and downregulation of glycolysis via AMPK signaling (16). Of note, in autoimmunity and various other inflammatory diseases, oxidative stress can disrupt the mitochondrial network integrity, compromise OXPHOS, deplete cellular ATP, and alter cellular metabolic pathways (17). In addition, the systemic and tissue microenvironmental changes seen in many disorders, including deficiencies in metabolites and nutrients as well as changes in oxygen levels can result in alterations in Treg function and stability via their metabolic reprogramming. In the absence of oxygen, the hypoxia-inducible factors (HIFs) orchestrate the transcription of an array of target genes that regulate metabolic pathways to adapt cells to low-oxygen environments by promoting glycolysis and suppressing mitochondrial respiration (18, 19). In line with the latter notion, studies have shown that hypoxia can impair Treg differentiation by promoting aerobic glycolysis by stabilizing one of the HIF isoforms (HIF1α) (20). Moreover, Yamamoto et al. provided further supporting evidence, showing that stabilization of both isoforms HIF1α and HIF2α in adult mice, through silencing of the prolyl hydroxylase domain 2 enzyme (PHD2), results in an impaired ability of Tregs to suppress either skin allograft rejection or *in vitro* responder T cell proliferation (21). However, despite the extensive literature to date, the molecular mechanisms that define Treg cell fate and plasticity and the interlinked processes of metabolism and redox homeostasis are still not well described in health and the changes seen in disease. This is of paramount importance given the fundamental role of impaired Treg function in an array of pathological conditions (22–25), with an increased Treg number and function reported in several mouse and human cancers (26–29), representing a major barrier to anti-tumour immunity. To gain an understanding of the integration of these processes the next sections review the key antioxidant and nutrient sensing pathways that determine Treg redox homeostasis and function and the impact of microenvironmental metabolic perturbations and nutrient availability.

## ROS production and antioxidant mechanisms

Tregs predominantly use fatty acid oxidation that generates acetyl-CoA to fuel the TCA cycle. In a series of enzymatic reactions, the TCA cycle generates the reducing equivalents NADH and FADH2 which are required to transfer electrons to the mitochondrial respiratory chain. The energy released from these oxidation-reduction (redox) reactions, along with the creation of a proton gradient, results in the synthesis of ATP by OXPHOS reactions. During this process, 0.2%–2% of the electrons leak out of the transfer and interact with O_2_ to produce ROS, such as superoxide or hydrogen peroxide (30, 31). Although most mitochondrial ROS results from OXPHOS, another three reactions during the TCA cycle generate ROS: pyruvate to acetyl-CoA (32, 33), α-ketoglutarate to succinyl-CoA (34, 35) and fumarate to succinate (36). Despite this, mitochondrial ROS levels can also be increased when there is disruption to the electron transport chain, resulting several pathological processes such as ageing, autoimmunity (17) and neurodegeneration (37).

Aside from the mitochondria, NADPH oxidases (NOXs) are also a source of ROS, with NOX2 described as a major ROS-generating enzyme in T cells (38). The negative impact of ROS in Tregs has been highlighted by studies reporting that NOX2 deficient Tregs exhibit higher suppressive capacity than wild-type Tregs both *in vitro* and *in vivo* (39, 40). This, therefore, highlights that the balance between production and consumption of ROS is an important factor in the development and function of Tregs. In line with this, and in the setting of chronic liver disease, recent work from our group has shown that Treg function and viability is negatively impacted by oxidative stress and increased intracellular ROS (41). Mechanistic insights revealed this to be secondary to an imbalance in redox homeostasis with an increased NOX-2 activity and dysregulated endogenous antioxidant signaling pathway.

The transcription factor NF (erythroid-derived 2)-like 2 (NRF2) is a basic leucine zipper transcription factor that serves as a master regulator of antioxidant defense against the cytotoxic effects of oxidative stress by regulating an array of detoxifying and antioxidant genes. Under quiescent conditions, NRF2 is retained and degraded in the proteasome by Kelch like ECH-associated protein 1 (Keap1) in concert with the E3 ubiquitin ligase Cullin 3 (Cul3) (42). Oxidative stress induces conformational changes in Keap1 that allow NRF2 release and translocation into the nucleus where it promotes the expression of phase II detoxifying enzymes and antioxidant proteins (43). Of note, NRF2 also targets the expression of several components of the glutathione (GSH) and thioredoxin (TRX)-1 antioxidant systems (44). Activated T cells, maintain the concentrations of intracellular ROS by using GSH (45) which triggers cytosolic ROS depletion either in a direct way or through enzymatic catalysis mediated by glutathione peroxidases (GPX). Cellular GSH content is largely determined by *de novo* synthesis, a process mediated by two ATP-dependent ligases, glutamate-cysteine ligase (GCL) and glutathione synthase (GS), as well as the availability of its constituent amino acids, cysteine, glycine and glutamate (46). TRX1 is also critical for Treg's resistance to oxidative stress. This enzyme scavenges reactive oxygen species and regulates other enzymes metabolizing H_2_O_2_ (47). Moreover, TRX1 maintains sustained surface expression of thiols as the first line of defense against ROS and is sensitive to proinflammatory stimuli, mainly tumor necrosis factor-α, in a nuclear factor-*κ*B-dependent fashion, which likely boosts Treg survival under inflammatory conditions (48).

## mTOR signaling in Tregs

Despite their role in the neutralization of excess ROS, the cellular antioxidant mechanisms can modulate Treg metabolic rewiring via engagement of the mammalian target of rapamycin (mTOR) pathway.

mTOR is a conserved serine/threonine kinase that senses and integrates diverse immune receptor signaling pathways and environmental cues to coordinately regulate metabolic programs that decipher T cell differentiation, proliferation, and function (49). mTOR is a component of two distinct multi-protein complexes termed mTOR complex 1 (mTORC1) and 2 (mTORC2) which share the catalytic subunit mTOR and the associated proteins Deptor and mLST8. PRAS40 and Raptor proteins are unique components of mTORC1, while mTORC2 is characterized by the expression of mSIN1 and Rictor (50). The upstream and downstream components of mTOR signaling differ in each complex. mTORC1 activity is mainly dependent on the small G protein RHEB (Ras homologue enriched in brain) which in turn is modulated by the tuberous sclerosis 1 (TSC1)–TSC2 complex (51, 52). This complex integrates positive and negative upstream signals transduced from the PI3/AKT and AMPK (AMP-activated protein kinase) pathways (53). An array of stimuli induces the PI3K/AKT pathway including TCR stimulation (54, 55) and co-stimulation signals mediated by CD28 or OX40 (56, 57), toll-like receptor signals (58, 59), cytokines (60, 61), growth factors and hormones (e.g., leptin and bioactive lipid sphingosine-1-phosphate-S1P) (14, 62). Besides PI3K/AKT dependent mechanisms, essential and non-essential amino acids can also activate mTORC1 via RAG GTPases (63). Moreover, other nutrients such as glucose and lipids can influence mTORC1 activation, however the mechanism for such regulation remains to be elucidated. During energy deprivation, AMPK downregulates mTORC1 directly by phosphorylation of Raptor and indirectly by phosphorylation of TSC2 (64, 65). Compared to mTORC1, regulation of mTORC2 is far less well understood. Studies in non-T cell lineages have postulated that its activity is modulated by PI3K signaling in response to growth factors and insulin which lastly mediates mTORC2-ribosome binding (66, 67). Moreover, other signals independent of PI3K enhance mTORC2 activity such as amino acid, glucose and glutamine starvation (68–70.) Following activation, mTORC1 drives c-MYC and HIF1α signaling via multiple mechanisms involving S6K1 and 4EBP1 to ensure induction of aerobic glycolysis and glutaminolysis necessary for conventional T cell activation and differentiation (71, 72). Meanwhile, mTORC2 activates AGC kinases, including Akt, SGK1 and PKCα, to promote cell survival, cell cycle progression and anabolism (73–75).

Observations regarding mTOR activity in Tregs have been complex and at times seemingly controversial. First, several studies revealed that the efficiency of Treg generation both *in vivo* and *in vitro* could be markedly enhanced in the presence of rapamycin (76–80). This result was further supported by genetic deletion of components of the mTOR signaling pathway (81–83). In addition, it was found that constitutive activation of the PI3K/Akt/mTOR network antagonized FoxP3 induction along with other Treg-signature transcripts (82, 84), which are essential in the generation and sustenance of the Treg lineage (85–87). Additionally, induced-Treg cell generation requires a metabolic switch to fatty acid oxidation via AMPK, thereby inhibiting mTORC1 signaling (88). In line with these results, studies demonstrated that negative regulation upstream of mTOR in Tregs is required for the maintenance of their suppressive function, thus indicating that the glycolytic metabolism induced by mTOR is dispensable for Treg cell function (28, 89–91). Despite these data, it seems paradoxical that other studies revealed that mTOR activity was increased in Tregs compared to Teff at steady state and promotes Treg proliferation (14, 92). According to these findings, Zeng et al. showed that Raptor/mTORC1 signaling coordinates cholesterol and lipid metabolic programs to support Treg proliferation, upregulation of the suppressive molecules CTLA-4 and ICOS, and functional fitness in part through inhibiting mTORC2 (92). Likewise, effector Tregs were characterized by increased mTOR activity, glycolysis, and effector molecule upregulation after robust TCR stimulation and in presence of a suppressive microenvironment (TGF-*β*) (93). Finally, Chapman et al. showed that *in vivo* inhibition of mTOR disrupts Treg suppressive function and leads to uncontrolled conventional T cell activation, especially associated with an excess of Th2 at mucosal tissues (94). Mechanistically, mTOR regulates the expression of the transcription factors IRF4 and GATA3 that are essential to activate the machinery involved in Treg-dependent suppression (95). Moreover, mTOR promotes mitochondrial fitness most likely via Raptor-mTORC1 as Raptor-deficient Tregs were not able to evade T helper responses in a colitis mouse model while Rictor-deficient Tregs showed normal mitochondrial activity and *in vitro* suppressive capacity (92). mTORC2 signaling by CD28 stimulation is required for Treg migration and involves the induction of the enzyme glucokinase (GCK) which is essential to maintain the glycolytic flux (96). These observations supported a key role for Raptor-mTORC1 pathway in establishing the fate and function of activated Tregs. However, the local environmental signals that may orchestrate mTOR tuning and hence the Treg function in health and the changes seen in various pathologies remain to be deciphered.

## Reciprocal regulation of mTOR and antioxidant mechanisms

**Figure 1:**
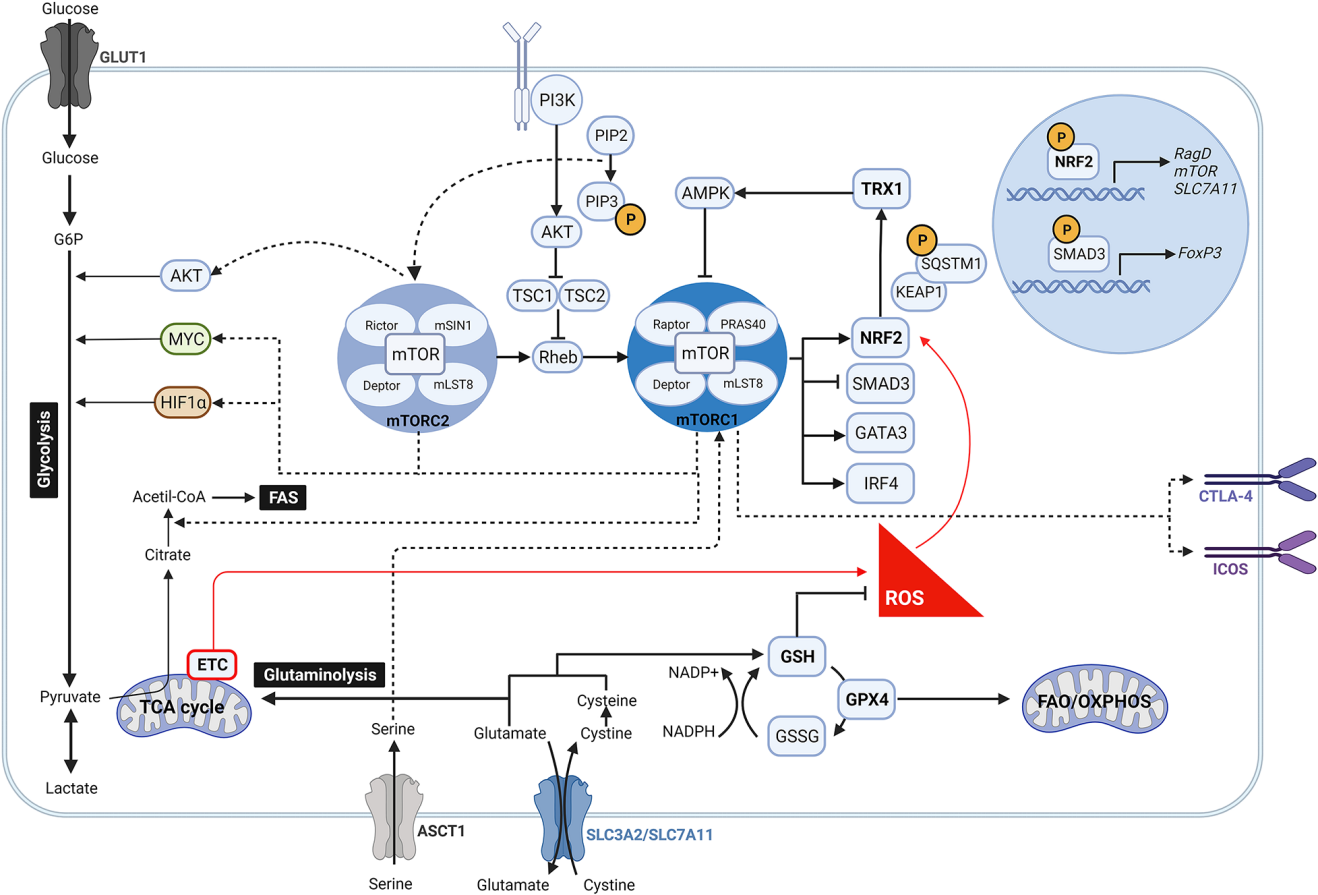
Crosstalk between mTOR pathway and anti-oxidant signalling in regulatory T cells. During oxidative stress, high levels of ROS induce the activity of NRF2 by disrupting its binding to KEAP1. Once NRF2 is released, it is translocated into the nucleus where it promotes the expression of mTOR, RagD and components of the PI3K/AKT pathway, which contribute to activate the mTOR complex signalling pathway. Likewise, increased mTORC1 activity may be link to an intracellular accumulation of serine due to an upregulated expression of its transporter ASCT1 by NRF2. Following activation, mTORC1 and mTORC2 are involved in many different signalling pathways. While mTORC2 modulates AKT, mTORC1 drives MYC and HIF1α downstream signalling so as to ensure aerobic glycolysis. mTORC1 reciprocally prompts NRF2 activity by phosphorylation of SQSTM1, inhibits the transcription of FoxP3 by SMAD3, and regulates the expression of the transcription factors IRF4 and GATA3. Moreover, mTORC1 is linked to the upregulation of CTLA-4 and ICOS, and to the induction of fatty acid biosynthesis programs. At the same time, NRF2 targets the expression of the glutamate/cystine transporter SLC7A11 that supports the GSH system and glutaminolysis. GSH boosts the removal of ROS which in turn reduce the activation of NRF2 and antagonize mTOR induction. Along this mechanism, NRF2 can also fine-tune mTOR activity via TRX1 that releases AMPK.

The antioxidant mechanisms GSH, NRF2 and TRX1 can regulate mTOR activity by different mechanisms (Figure 1). NRF2 directly modulates mTOR transcription by binding to the mTOR promoter (97). Although it has also been reported that NRF2 can upregulate mTOR activity indirectly by increasing the expression of RagD, a protein known as an activator of mTOR or by modulating activity of components of the PI3K/Akt pathway (63, 98). In line with these observations, Klemm et al. showed an increased phosphorylation of mTOR and of the mTOR target p-S6 in Tregs of Foxp3^cre^ mice with constitutive NRF2 activation. Their results suggest that enhanced mTOR signaling led to elevated glucose uptake that impacts Treg long term survival and lineage stability with reduced FoxP3 expression and immunosuppressive function (99), supporting the role of mTORC1 as a negative regulator of Treg cell function. A previous study also showed that absence of NRF2 in donor T cells enhanced persistence of Tregs and reduced systemic inflammation in graft-vs.-host disease mice (100). Additionally, Noel et al. demonstrated that T cell-specific activation of NRF2 increases the number of Tregs and improves Treg-mediated suppression of inflammation in an ischemia reperfusion-induced acute kidney injury mouse model (101).

More recent data have outlined the role of NRF2 along with other antioxidant mechanisms in controlling mTOR activity and Treg function. To diminish mTOR activity, GSH scavenges ROS to control NRF2 activation which decreases the amino acid transporter ASCT1 and therefore serine uptake (102). In line with this, intracellular accumulation of serine has been shown to result in Treg proliferation at the cost of FoxP3 expression and suppressive function (102). As GSH controls NRF2 activation, which in turn releases TRX1 that inhibits AMPK (103), it appears evident that Tregs also use GSH to indirectly modulate mTOR signaling (Figure 1). The antagonistic effect of TRX1 in controlling mTOR activation is further supported by Chakraborty et al., who did not report an increase in the quantity and suppressive quality of induced Tregs generated by using a recombinant Trx (104).

As the cellular antioxidant mechanisms control mTOR activity, mTOR can reciprocally regulate them. In this context, mTORC1 enables NRF2 release from its principal negative regulator KEAP1 by phosphorylation of sequestosome 1 (SQSTM1), thus inducing NRF2 stabilization and translocation into the nucleus (105–108), where it drives cystine/glutamate antiporter solute carrier (SLC)7A11 expression. This transporter together with SLC3A2 (or CD98) are responsible for maintaining the cysteine intracellular pool, which in addition to protein synthesis, is essential to produce GSH and boost redox balance (109–111). In an elegant study Procaccini et al. described this mechanism as a key determinant of Treg proliferative capacity in patients with relapsing-remitting multiple sclerosis (RRMS) (112). In their study they showed a decreased expression of SLC7A11 by patient-derived Tregs, linked to a dysregulated NRF2 signaling, which was rescued upon treatment of these patients with dimethyl fumarate (DMF), small molecule inducer of NRF2 licensed for the treatment of RRMS (112). These findings highlight NRF2 as an indispensable checkpoint to support redox homeostasis and determine Treg cell expansion (12).

## Lipid metabolism and mitochondrial integrity

The antioxidant mechanisms can directly modulate Treg cell outcome regardless of mTOR activity. Upon activation, Tregs markedly reprogram lipid synthesis and fatty acid oxidation in support of their expansion and suppressive function (88, 113). In fact, several studies have suggested that Tregs rely on fatty acid synthesis and oxidation to survive and proliferate in the hostile tumor environment (114, 115). However, this mechanism potentially enhances generation of toxic lipid peroxides and subsequent ferroptosis, that can impair cellular redox homeostasis. One of the members of the antioxidant GPX family, GPX4, directly reduces membrane phospholipid hydroperoxides and oxidizes lipoproteins by using GSH as a cofactor to maintain redox balance under active lipid metabolism (116–118). Along these lines, a recent study demonstrated that inducing ferroptosis of intra-tumoral Treg cells by GPX4 deficiency disrupted mitochondrial fitness and enhances interleukin (IL)-1β production, which potentiates Th17 responses (119).

## Iron metabolism and PDK1

Iron homeostasis is tightly linked to lipid metabolism and ROS production. In short, excess iron ions (Fe^2+^) convert the hydrogen peroxide (H_2_O_2_), produced by mitochondrial respiration, into the toxic free hydroxyl radical (OH) through Fenton/Haber-Weiss reactions, which in turn need electrons from lipids resulting in an accelerated lipid peroxidation and ferroptosis (120). The phosphoinositide-dependent kinase 1 (PDK1) has been shown to play a key role in maintaining iron-dependent ROS levels and the suppressive function of Tregs. A recent study described that PDK1 inhibits MEK-Erk signaling and thus expression of the transferrin receptor protein 1 (CD71) and iron uptake (121). Apart from iron metabolism, PDK1 regulates multiple signaling pathways including the canonical NF-kB pathway and Akt/mTOR pathways (122). Indeed, the previous study showed that PDK1-deficient Tregs exhibit downregulation of the mTORC1 pathway and an altered mitochondrial metabolism which likely explains the impaired Treg function. Moreover, Hyunju Oh et al. reported that PDK1 controls Treg signature gene expression by regulating the canonical NF-κB pathway and that Treg-specific deletion of PDK1 in mice results in a severe systemic inflammation, accompanied by reduced number and suppressive function of Tregs (123).

## Lactate and pyruvate metabolism

Besides the antioxidant mechanisms, different metabolic pathways may also play a key role in the resistance of Tregs to oxidative stress in certain environments. As already outlined in previous sections, Tregs are less dependent on glycolysis and largely use OXPHOS to fulfill their bioenergetic demands (12, 13). In the setting of metabolic stress, as seen in solid tumors, the alterations in the availability of nutrients, such as glucose, glutamine, and tryptophan and the enrichment in lactic acid and kynurenines (124), have an impact on the tumour immune compartment. In this regard, extensive literature reports the negative impact of this metabolic environment on the cytotoxicity of effector T cell responses, which in-essence are dependent on glycolysis (125–127). In contrast, intra-tumoral Treg's metabolic reprogramming in this environment is evident by the use of alternative metabolites, such as lactic acid, to support their survival and function (128). Indeed, FoxP3 has been shown to favor oxidation of L-lactate to pyruvate while quenching glycolysis by downregulation of Myc (114). In addition to their role as substrates to produce ATP, lactate and pyruvate have also been described to have potent roles in resistance against oxidative stress. In this regard, in their study, Tauffenberger et al. show that oxidative metabolism of L-lactate, and to a lesser extent pyruvate, boosts mitochondrial activity and promotes a moderate increase in ROS production that is sufficient to induce NRF2 activity, PI3k/AKT and mTOR signaling and thereby support cell survival (129). However, this mechanism has been described in an *in vitro* model, and it remains to be elucidated whether lactate activates detoxifying mechanisms under oxidative stress conditions *in vivo*.

## Therapeutic potential: harnessing the ROS-metabolism interplay in Tregs for immunoregulatory therapies

The molecular pathways described above provide multiple opportunities to finely tune ROS homeostasis and metabolic programs in Tregs by targeting signaling cascades, enzymes and metabolites. In so doing, we anticipate it will be possible to either boost Treg function (to promote tolerance in autoimmunity or transplantation) or inhibit it (in cancer or chronic infections).

For instance, by specifically targeting a ROS-producing enzyme, Trevelin et al. observed that the depletion of Nox2 within Tregs in murine heart transplant recipients promoted the engraftment of allogeneic grafts. Mechanistically, Nox2-deficient Tregs exhibited notable changes: (1) increased expression of chemokine receptors CCR4 and CCR8, promoting their migration toward the allograft; (2) heightened capacity to suppress the proliferation of CD8+ T effector cells and the production of interferon-gamma (IFN-γ); and (3) improved survival (40). The understanding of the metabolic pathways underpinning the heightened functionality of Nox2-deficient Tregs, however, remains to be elucidated.

mTOR is another target influencing Treg metabolism that has been explored for therapeutic purposes, although its downstream effect on Treg function appear to be highly context dependent and far from being well understood. Thus, several studies have reported that mTOR inhibition, using Rapamycin or Everolimus (EVR), facilitates the selective *ex vivo* expansion of Tregs to generate cell therapy products for adoptive transfer into transplant recipients or autoimmune patients (130–132). To uncover the underlying mechanisms of mTOR inhibition in this context, Gedaly and colleagues conducted a metabolic characterization of autologous Tregs from renal transplant patients during the initial phase of their *ex vivo* expansion in the presence of Rapamycin or EVR (133). During the first 5 days of expansion, both compounds reduced the glycolytic rates and promoted an OXPHOS energy profile in Tregs, although the EVR-treated Tregs showed a reduced OXPHOS activity that was associated with diminished mTORC2 signaling and slow initial expansion rates. The distinct bioenergetic profiles and the relative reliance on OXPHOS and glycolysis during the expansion of Tregs and Tconv cells contributed to the selective expansion of Tregs. Despite such a study, several questions remain to be addressed, such as the metabolic characteristics and redox state of Tregs during expansion and the impact of the microenvironment on the infused cells. This information would lend itself to optimization of the current protocols/process to ensure the expansion of a product that is stable/ functional and resilient to the stressors of the microenvironment, once injected.

Of note, the difficulties of standardizing Treg manufacture protocols, together with the known efficacy advantages of conferring antigen-specificity to Treg products (134), have prompted the use of the chimeric antigen receptor (CAR)-engineered technology. Human leukocyte antigen (HLA)-A2-targeted CAR-Tregs have been shown to efficiently prevent graft-vs.-host disease and skin graft rejection (135–138), and are currently in clinical trials in kidney and liver transplantation. There is limited information, however, concerning their metabolic requirements, and whether their function, lineage stability, proliferative potential, and survival can be further optimized by modifying metabolic programs.

Examples of how this could be achieved are from lessons learnt from the CAR-T oncology field, where genetic modifications and extracellular metabolite supplementation strategies have demonstrated the ability to influence mitochondrial metabolism, extend the longevity of CAR-T cells, and counteract their exhaustion. For instance, incorporating the co-stimulatory endodomain 4-1BB into CAR-T molecules has been shown to promote the upregulation of the peroxisome proliferator-activated receptor (PPAR)-γ coactivator (PGC)-1α via p38-mitogen-activated protein kinases (MAPK) pathway. This leads to mitochondrial fusion and biogenesis, significantly boosting T cell respiratory capacity (139). Such CAR-T configurations have proven clinically effective, enhancing antitumor immunity and long-term T cell survival in leukemia patients (140, 141). Notably, a recent publication has underscored the benefits of including PGC-1α in the CAR structure (142). Alternatively, Ligtenberg et al. described a different strategy involving the co-expression of catalase with the CAR construct (CAR-CAT) in adoptive T cells. This approach neutralizes H_2_O_2_ and bolsters cellular antioxidative capacity, thereby sustaining CAR-T viability and antitumor function under oxidative stress (143). The pre-conditioning employed to facilitate the engraftment of CAR-T cells, which results in increased levels of IL-2, IL-7, IL-15, and IL-21 in the circulation, offers additional opportunities to influence metabolism. IL-2 promotes glycolysis through the Akt-mTOR pathway, and differentiation of CD8+ T cells into an effector memory phenotype (144); IL-7 enhances memory CD8+ T cell formation by inducing the expression of glycerol channels and increasing triglyceride synthesis (145); IL-15 increases the spare respiratory capacity and oxidative metabolism rate by enhancing mitochondrial biogenesis and CPT1a (Carnitine Palmitoyl transferase 1a) expression, which results in a stem cell memory T cell phenotype (146); IL-21 modulates the induction of central memory T cells and acquisition of the exhaustion phenotype, increasing antitumor efficacy in an fatty acid oxidation-dependent manner (147). Furthermore, pre-treatment of CAR-T cells with compounds that modulate the PI3K-AKT-mTOR pathway, such as PI3K inhibitors, Rapamycin, AMPK activators like Metformin or MAPK inhibitors, have demonstrated the potential to enhance the fate and potency of CAR-T cells. This approach helps preserve memory-like characteristics by overall promotion of fatty acid oxidation and mitochondrial biogenesis (148–152). Finally, the use of mitochondria-targeted antioxidants offers a novel strategy to counteract cytotoxic T cell exhaustion. Antioxidant treatment effectively reduces the levels of ROS generated by chronically stimulated T cells and mitigates the decline in mitochondrial fitness (153).

In addition to these mechanisms, therapeutically shaping the local microenvironment to mitigate oxidative stress and foster redox homeostasis offers additional opportunities to protect/boost Tregs. In this context, the gut microbiota appears to be key. Thus, various metabolites produced by bacteria have been linked to immune homeostasis and systemic inflammation (154, 155). Some of them have a strong effect on Tregs: microbial short-chain fatty acids and tryptophan promote the suppressive function of Tregs (156, 157). On the other hand, certain bacteria possess the ability to generate and counteract reactive oxygen species (ROS), thereby regulating the host's redox state (158–160). Currently, innovative therapies are in development to modulate the gut microbiota, particularly in gastrointestinal diseases characterized by excessive ROS production, such as inflammatory bowel disease and colorectal cancer (161, 162). Further studies are needed to understand whether these approaches hold promise in supporting redox homeostasis and metabolic fitness of Tregs.

## Concluding remarks

The mechanisms underlying the complex and interlink networks between redox homeostasis and metabolism as determinants of Treg fate are ill defined. In this review, we summarize the signaling and metabolic pathways that are known to be fine-tuned by oxidative stress in Tregs with special attention to the antioxidant mechanisms that coordinate many of the signaling pathways that contribute to metabolic rewiring, ultimately leading to Treg survival and function. In this regard, Tregs from healthy subjects express and secrete high levels of antioxidants, specially TRX1, indicating a higher endogenous antioxidant capacity than Tconv (121, 122). However, it is still controversial whether Tregs exhibit greater resistance to oxidative stress-induced cell death, particularly in the context of an oxidant/antioxidant imbalance (122, 123). Indeed, further insight is needed into the precise modulatory switches that may delineate Treg fate under steady state and the effect of oxidative stress and various pathological processes. It is also important to highlight the main challenges involved in the study of these interlinked networks. This includes the complexity of environmental cues: factors such as hypoxia, nutrient deprivation and the presence of a vast array of extracellular metabolites, all affecting intracellular regulatory mechanisms. Additionally, the heterogeneity of Tregs, activation states and interactions with other immune cells, will inevitably be governed by these mechanistic processes. Therefore, the future will see an integration of not only systems-based approaches, but computational and mathematical modeling of these cellular networks. The application of these approaches will advance our understanding of these complex networks and interactions as determinants of Treg function, inevitably leading to therapeutic targets in several conditions governed by aberrant immune responses.
